# From Microbial Dynamics to Functionality in the Rhizosphere: A Systematic Review of the Opportunities With Synthetic Microbial Communities

**DOI:** 10.3389/fpls.2021.650609

**Published:** 2021-06-03

**Authors:** Olga Marín, Bernardo González, María Josefina Poupin

**Affiliations:** ^1^Laboratorio de Bioingeniería, Facultad de Ingeniería y Ciencias, Universidad Adolfo Ibáñez, Santiago, Chile; ^2^Center of Applied Ecology and Sustainability (CAPES), Santiago, Chile

**Keywords:** SynCom, plant growth promoting bacteria, holobiont, plant microbiome, rhizosphere, biocontrol, PGPR, core microbiome

## Abstract

Synthetic microbial communities (SynComs) are a useful tool for a more realistic understanding of the outcomes of multiple biotic interactions where microbes, plants, and the environment are players in time and space of a multidimensional and complex system. Toward a more in-depth overview of the knowledge that has been achieved using SynComs in the rhizosphere, a systematic review of the literature on SynComs was performed to identify the overall rationale, design criteria, experimental procedures, and outcomes of *in vitro* or *in planta* tests using this strategy. After an extensive bibliography search and a specific selection process, a total of 30 articles were chosen for further analysis, grouping them by their reported SynCom size. The reported SynComs were constituted with a highly variable number of members, ranging from 3 to 190 strains, with a total of 1,393 bacterial isolates, where the three most represented phyla were Proteobacteria, Actinobacteria, and Firmicutes. Only four articles did not reference experiments with SynCom on plants, as they considered only microbial *in vitro* studies, whereas the others chose different plant models and plant-growth systems; some of them are described and reviewed in this article. Besides, a discussion on different approaches (bottom-up and top-down) to study the microbiome role in the rhizosphere is provided, highlighting how SynComs are an effective system to connect and fill some knowledge gaps and to have a better understanding of the mechanisms governing these multiple interactions. Although the SynCom approach is already helpful and has a promising future, more systematic and standardized studies are needed to harness its full potential.

## Introduction

One of the most relevant discoveries in biological research of the past couple of decades is the role that host-associated microbial communities (microbiomes) have in health (Berendsen et al., 2012; Gallo and Hooper, 2012; Mendes et al., 2013; Haney et al., 2015; Pieterse et al., 2016), nutrition (Hacquard et al., 2015), growth (Lugtenberg and Kamilova, 2009), and even behavior throughout the plant and animal kingdoms (Wagner et al., 2014; Vuong et al., 2017; Lowry et al., 2018). Nevertheless, the mechanisms underlying individuals' interactions from different taxonomic domains are generally challenging to assess, and the rhizosphere is not an exception. Indeed, the rhizosphere is a perfect example of an environment where it is possible to find highly complex intradomain and interdomain interactions. The challenges are usually associated with the vast biodiversity that is present in this environment (Durán et al., 2018; Xiong et al., 2020), the edaphic factors and physical structures that make up countless microniches available for microbial colonization (Allard-Massicotte et al., 2016; Robertson-Albertyn et al., 2017; Howard et al., 2020; Kong et al., 2020), and the chemical richness with its spatiotemporal variety as a consequence of the organisms that interact under and above this realm (Chaparro et al., 2014; Staley et al., 2017; Vives-Peris et al., 2020).

The interplay between hosts and their associated microbiomes affects the ontogeny of the partners and is also seen as a fundamental basis of microbial ecology and evolution (Rausch et al., 2019; Batstone et al., 2020). Likewise, the role of microbiomes in the adaptation and evolutionary processes of plants is under analysis (Rosenberg and Zilber-Rosenberg, 2016; Hawkes et al., 2020).

Over the years, many different techniques and procedures have been used to decipher plant–microbe and microbe–microbe interactions in the rhizosphere. In a broad sense, there are two ways to address the environmental–molecular biology topic questions. Reductionist approaches seek to control as many experimental factors as possible, usually analyzing plant–microbe interactions where the players are well-known (Liu et al., 2019). Good examples of these approaches are those studying interactions, such as plant–mycorrhizal associations (Krajinski and Frenzel, 2007; Nadeem et al., 2014), plant–pathogen protection (Pieterse et al., 1998; Pascale et al., 2020), the interactions between plants with symbiotic nitrogen-fixing (Ferguson et al., 2010; Sulieman et al., 2015), or plant growth–promoting rhizobacteria (Poupin et al., 2013; PGPRs, Pinedo et al., 2015; Timmermann et al., 2019). In contrast, holistic approaches aim to study the plant microbiome as a whole (holobiont), focusing on diminishing interferences to reduce environmental variation and elucidate how it operates in its natural environment (Fang and Casadevall, 2011; Tecon et al., 2019). Thus, different technologies and protocols are more or less well-fitted to each of these approaches; while the reductionist usually depends on culture-dependent methodologies, the holistic typically relies on culture-independent techniques such as high-throughput genomic (Raes et al., 2011). These two approaches are required to address microbial ecology issues as microbiomes operate as a whole or with subsets of them, but at the same time, each of their members is indeed an individual organism that may exert particular effects in plants. Unfortunately, holistic approaches likely stay in the first step of top-down analyses, and the reductionist approaches remain only in the earlier steps of a bottom-up exploration. Synthetic microbial communities (SynComs) are consortia designed to test hypotheses and to mimic, to some extent, the role of a particular microbiome and have received particular interest in recent years (Vorholt et al., 2017; reviewed in de Souza et al., 2020). Here, a systematic review of the literature on SynCom was performed to identify the overall rationale, design criteria, experimental procedures, SynCom characteristics, and outcomes of *in vitro* or *in planta* tests using SynComs in the rhizosphere. Additionally, this information was used to compare different approaches (bottom-up and top-down) to study the microbiome's role in the rhizosphere. Finally, the need for more systematic and standardized studies with SynCom is discussed. This will allow us to fill and connect some knowledge gaps, perform statistical meta-analyses, and better understand these interactions, where microbes, plants, and the environment are players of a multiscale and complex system.

## Methods

### Search Strategy

PubMed, Web of Science, and Scopus databases were browsed for eligible published articles up to November 2020, using specific words and terms related to rhizosphere microbial communities, plant growth–promoting traits, and *in vitro* and *in planta* assays. Twenty-two different combinations of the following terms were used: rhizosphere, root; reductionist, representative; synthetic; microbial, bacterial; community, communities; assembly, assemblage; consortium, consortia; and the abbreviations PGPR and SynCom. First, PubMed searches were carried out, and the five searches that retrieved eligible articles were repeated in Web of Science and Scopus to guarantee adequate coverage, providing six additional publications. These searches included the following terms: rhizosphere, root, microbial, SynCom, synthetic, consortia, community, communities, and assemblage.

### Selection Criteria

The inclusion criteria were as follows: (1) only research articles, excluding reviews, opinions, and case reports; (2) use of SynComs or mixed inoculations of at least three different bacterial strains/members, excluding use of indigenous, natural, or wild microbial communities (WildCom); (3) plant-related application; and (4) rhizosphere-related microbiota. Only articles in English and published from 2015 were included. The selection of this frame time is supported with the appearance of the seminal article of Bai et al. (2015), where the authors cultivated representative microbiome SynComs of the natural *Arabidopsis* leaf and root microbiome. A few additional articles were identified from references within these first selected articles or from relevant reviews related to the topic. After the application of all these criteria and a more thorough revision and filtering process, a total of 30 articles were finally selected. The filtering process results are indicated in a PRISMA diagram (Moher et al., 2009; Supplementary Figure 1). Finkel et al. (2019, 2020), and Ramirez-Villacis et al. (2020) reported the same SynCom with 185 members. To avoid a bias in the results, this SynCom was incorporated only once in the microbial taxonomic analyses, but the three articles were included in the rest of the analyses.

### Data Extraction

The data extracted from the articles consisted of authors, year, country, title, journal and impact factor, keywords (if any), plant model, research area and main objectives, SynCom members, SynCom assemblage procedures including culture conditions, complementary methods (if relevant), and main results. Some metadata on the retrieved articles is available in Supplementary Table 1. The taxonomic classification of each isolate was that which was referenced in each article. Nevertheless, when discrepancies were observed, the taxonomic identification was checked in the Taxonomy Browser of NCBI. In some cases, a taxonomic rank was not indicated in the reports; in that case, the term “unclassified” was used. The complete database generated in this work (with the isolates and taxonomic classification) is included in the Supplementary Table 2.

## Results

### General Results From the SynCom Literature Analysis

The combination of keywords and terms led to a set of 30 articles dealing with relatively narrow plant research areas (Supplementary Table 1), being the most reported topics, plant defense system, biocontrol and sustainable agriculture, abiotic stress, PGPRs, phytohormones, plant–microbe, and microbe–microbe interactions. Microbiome assembly and microbiota recruitment, bioremediation, experimental evolution, and ecology were also part of the retrieved articles.

**Figure 1:**
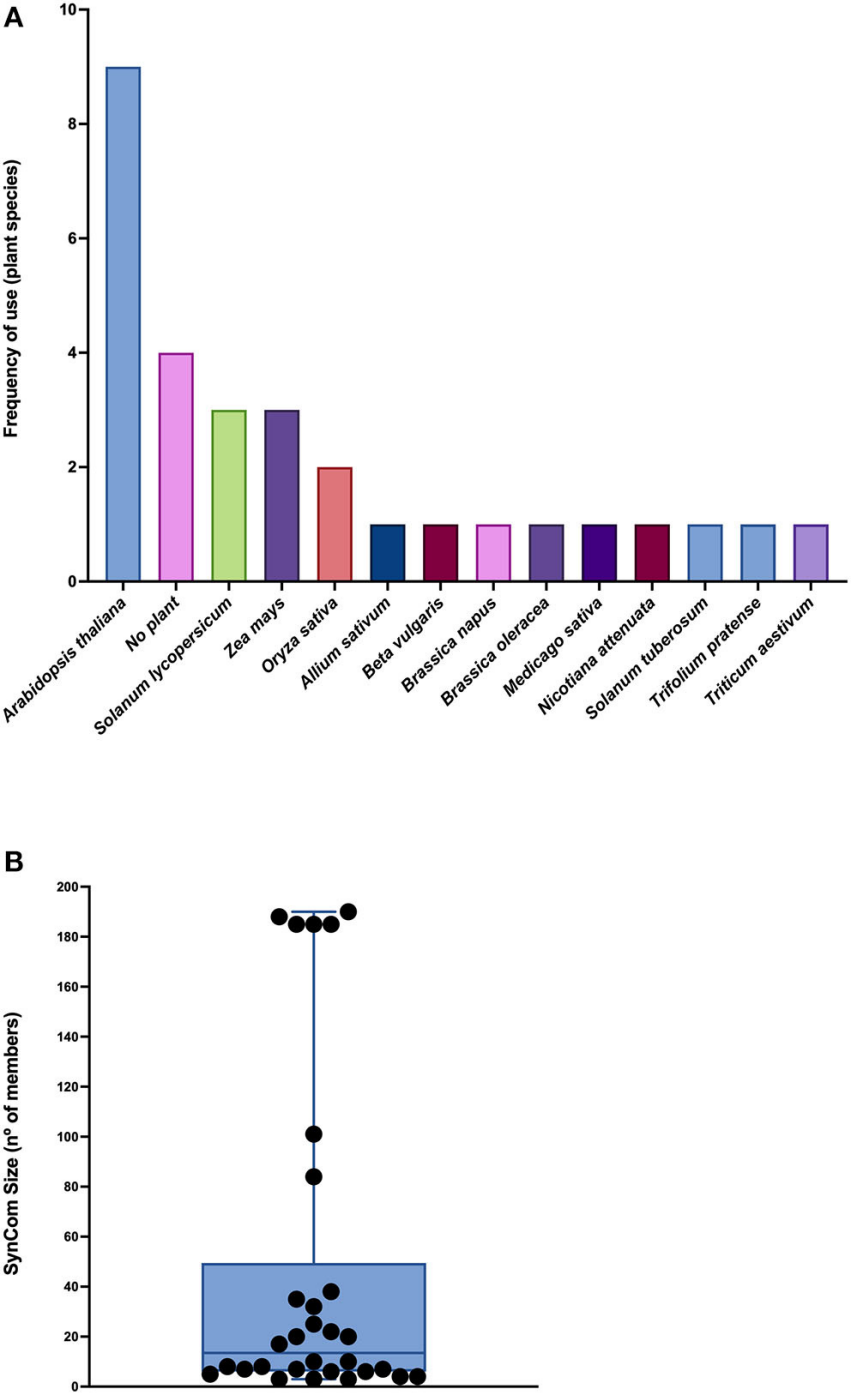
Plant models and SynCom sizes reported in the literature. **(A)** Frequency of use of plant species in the selected articles. **(B)** Box plot showing the number of members used in SynComs. Each dot represents an article. The horizontal line corresponds to the median.

Even though *Arabidopsis thaliana* was the most common plant model employed (nine of the 30 articles), several other plant models, mostly crops, were utilized in these studies (e.g., *Allium sativum, Brassica oleracea, Beta vulgaris, Oryza sativa, Solanum lycopersicum, Solanum tuberosum, Triticum aestivum*, and *Zea mays). Brassica napus, Nicotiana attenuata, Medicago sativa*, and *Trifolium pratense* were also used, and four articles did not include any plant model, as they consider only *in vitro* studies (Supplementary Table 1; Figure 1A).

Little standardization was observed throughout the methodologies and protocols from the articles reviewed. Different culture media, terminology (e.g., inoculum, mix, and consortium), optical density settings, and variable positive and negative controls were detected. There were only a couple of studies in which the effects of no inoculation, single-strain inoculation, and multistrain inoculation were tested simultaneously (Tsolakidou et al., 2019; Zhuang et al., 2020), and raw data were not always available. Therefore, it was impossible to perform a quantitative and statistical meta-analysis based on numerical data retrieved from the experiments.

### SynCom Design, Procedures, and *in silico, in vitro*, and *in planta* Studies

The main criteria or conditions detected in the design of a SynCom were two: (i) use microorganisms that can be specifically tracked by 16S rRNA gene amplicon analysis or an equivalent technique; i.e., the sequence must be available and not be too similar to other microorganisms; (ii) microorganisms must be culturable (Bai et al., 2015; Voges et al., 2019).

Regarding the bacterial culture media employed, Luria–Bertani (LB) and tryptic soy broth/tryptic soy agar (TSA) were the most used ones to grow bacteria, and Murashige and Skoog (1962) for plants. Depending on the objectives and length of the experiments, different growth systems were utilized. Agar was typically employed to germinate seeds, and when plants reached a time by which transplantation can be done, diverse soils and soil-like substrates were reported. For instance, Lebeis et al. (2015) carried out germination of seeds on MS agar and then transplanted the seedlings to fine soil mixed in a 2:1 ratio with steamed and autoclaved sand; Tsolakidou et al. (2019) carried out *in vitro* experiments on potato dextrose agar, TSA, and MS agar media and opted for a sterilized peat-based potting substrate for parallel tests; Castrillo et al. (2017) instead only employed a soil system based on a collected top soil from a site free of pesticide and fertilizer and mixed 2:1 with autoclaved sand to improve drainage. For microbial effects on germination, in Moronta-Barrios et al. (2018), seeds were grown in liquid LB medium, and 4 days after germination, individual seedlings were transferred to tubes containing semisolid, half-strength Hoagland solution to study the resultant inoculated-plant phenotype. Among the plant-growth systems, some others were also utilized, such as a hydroponic system (Voges et al., 2019); the FlowPot, which includes an autoclaved and a washed substrate such as peat (Durán et al., 2018; Kremer et al., 2018); or the Litterbox system, which uses zeolite covered by a polydimethylsiloxane sheet (Miebach et al., 2020). Each of these systems was selected to control different variables and to pursue different objectives. For instance, hydroponic systems were usually used to overcome problems arising from the heterogeneity of soils and soil substitute substrates (Voges et al., 2019).

Depending on the experimental objectives or questions to be addressed and concerning the presence of microorganisms, studies were carried out under sterile, axenic, gnotobiotic, or holoxenic conditions. While the first condition is free of any living microorganism (germ-free), the second one means “free of strangers” and may contain only deliberately inoculated microorganisms (Luckey, 1963). The gnotobiotic condition means an environment in which all the organisms, including microorganisms, are known by the investigator, whereas the last one implies undefined microbiota (Miebach et al., 2020).

The reported SynComs were constituted with a highly variable number of members, ranging from 3 to 190 strains, 13.5 being the median (Supplementary Table 1; Figure 1B); all of them except the one designed by Durán et al. (2018) comprised only bacterial populations. While the larger SynComs were intended to mimic the indigenous communities, spanning the phylogenetic tree as a holistic approach (e.g., Ramirez-Villacis et al., 2020), smaller consortia were built with a reductionist rationale focusing on each community member for its already known or presumed traits or influences (e.g., Zhuang et al., 2020). The selection of the strains was generally related to the following purposes: (i) to represent specific microbial functions or traits (e.g., phosphate solubilization; Zheng et al., 2019); (ii) to represent a specific taxonomic arrangement (e.g., the core microbiome of a specific plant species; Toju et al., 2018, 2020); (iii) based on interaction networks (e.g., biocontrol and pathogen resistance; Poudel et al., 2016; Durán et al., 2018; Kong et al., 2018); and (iv) to combine strains with previously known functions (e.g., Santhanam et al., 2019).

### SynComs Taxonomic Compositions

**Figure 2:**
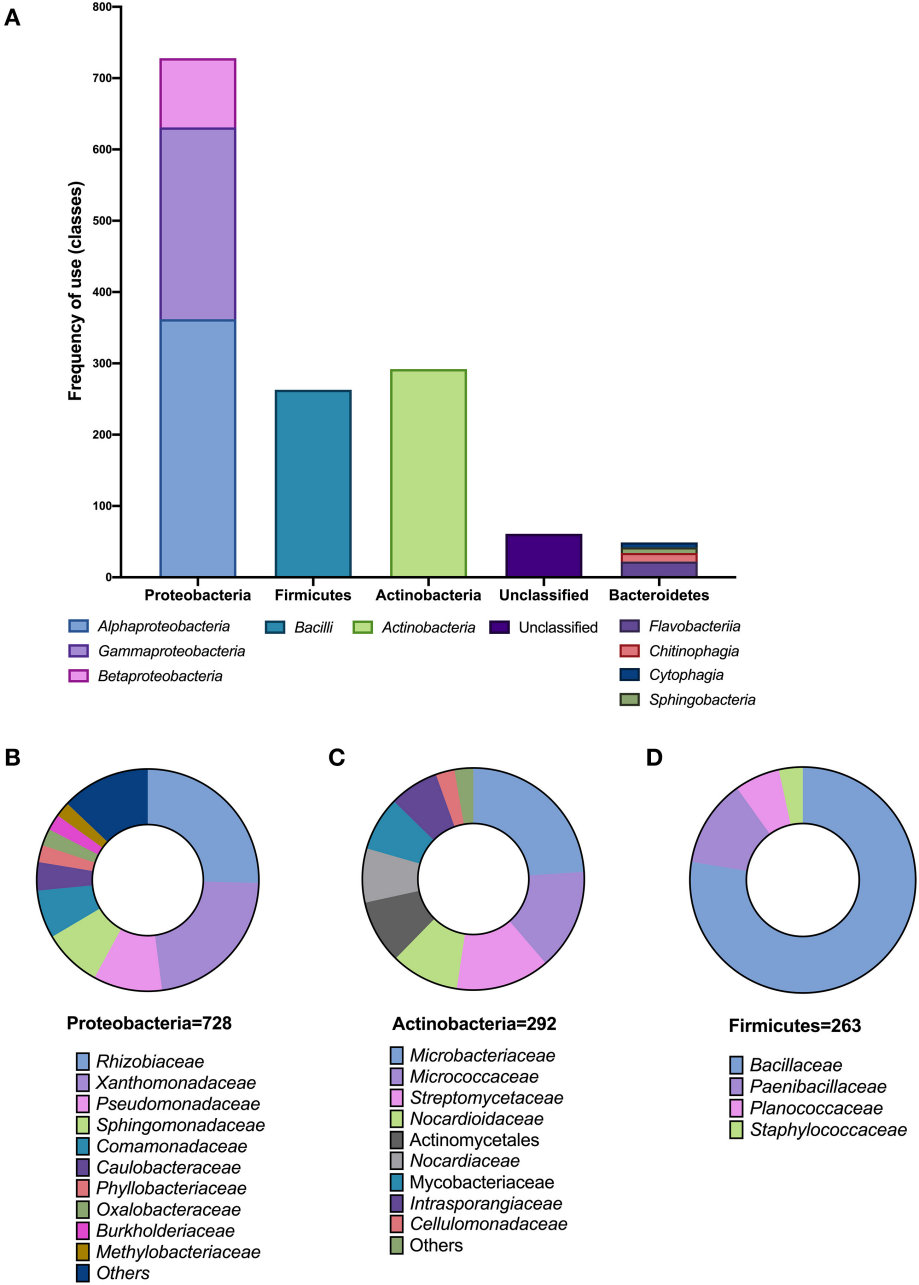
Taxonomic analysis of SynCom compositions. **(A)** Frequency of use (number of members) in different classes within each phylum in the reported SynComs. Families of the members of SynCom of the phyla Proteobacteria **(B)**, Actinobacteria **(C)**, and Firmicutes **(D)**.

From a total of 1,393 isolates listed in all analyzed SynComs, the three most represented phyla were Proteobacteria (728), Actinobacteria (292), and Firmicutes (263). Only 49 isolates were Bacteroidetes, and 61 were reported as unclassified (Figure 2A). Within Proteobacteria, the most represented families were *Rhizobiaceae* (185) and *Sphingomonadaceae* (62) from the Alphaproteobacteria; *Comamonadaceae* (51) from the Betaproteobacteria, and *Xanthomonadaceae* (164) and *Pseudomonadaceae* (73) from the Gammaproteobacteria classes (Figure 2B). For Actinobacteria, most isolates belonged to the families *Microbacteriaceae* (70), *Micrococcaceae* (43), and *Streptomycetaceae* (40) (Figure 2C). In the case of Firmicutes, 77.6% belonged to the *Bacillaceae* family (Figure 2D).

**Figure 3:**
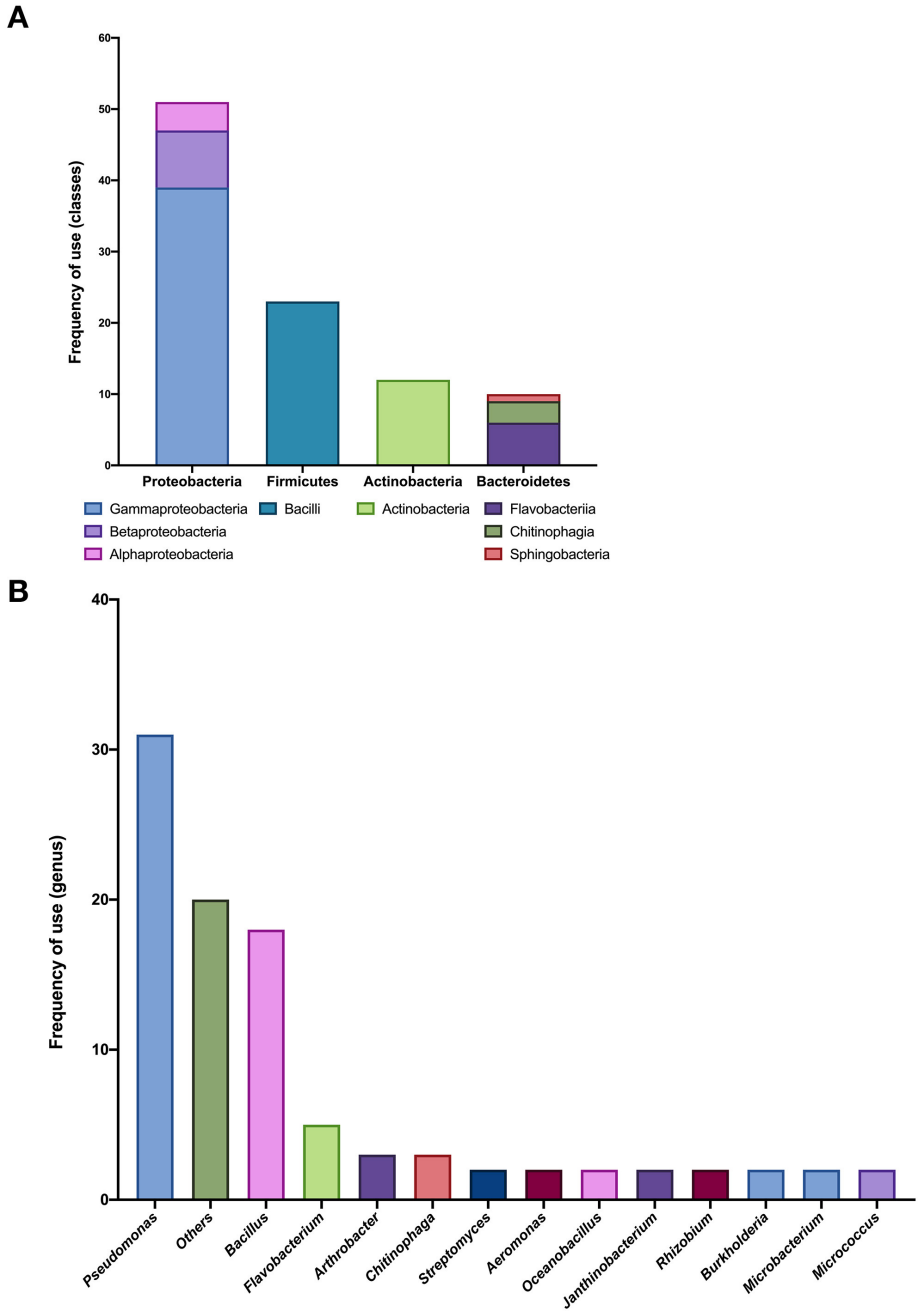
Taxonomic analysis of small SynCom compositions. **(A)** Frequency of use (number of members) in different classes within each phylum from 15 reported small SynComs. **(B)** Frequency of use (number of members) in different genera from 15 reported small SynComs.

Fifteen SynComs with up to 10 members were reported (small SynComs), with a total of 33 different genera (Figure 3), again with the phyla Proteobacteria, Firmicutes, and Actinobacteria as the most represented (Figure 3A). Within the Proteobacteria phylum, the Gammaproteobacteria class was the most reported (39 times), followed by the Betaproteobacteria (eight times), and none of the isolates were unclassified (Figure 3A). At the genus level, *Pseudomonas* (32.3%) and *Bacillus* (18.8%) were the more frequently used (Figure 3B). Only 55 isolates were reported at the species level, with *Pseudomonas fluorescens, Pseudomonas frederiksbergensis*, and *Bacillus megaterium* reported more frequently (Figure 3B).

## Discussion

### SynComs to Narrow the Gap Between Bottom-Up and Top-Down Strategies

**Figure 4:**
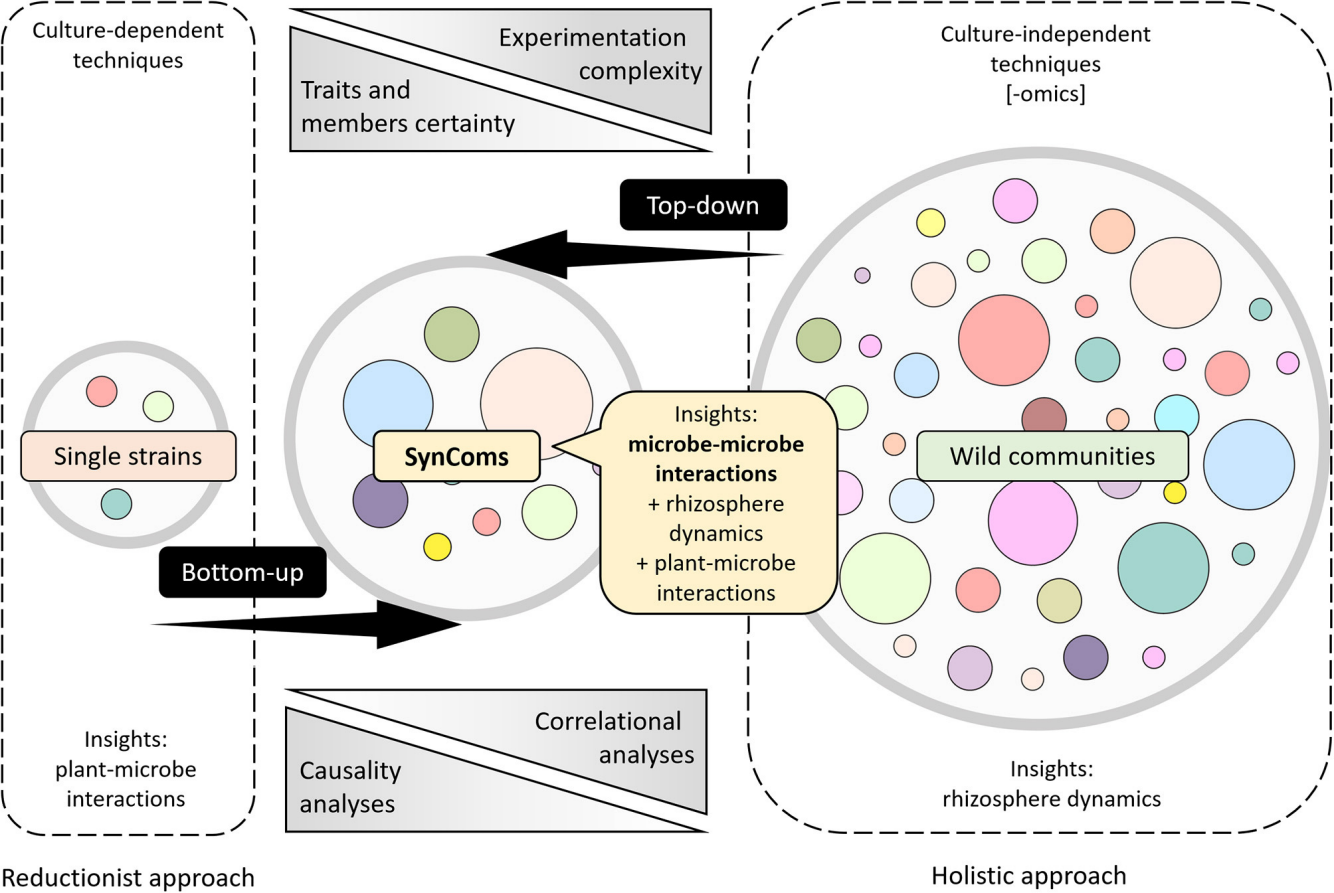
Different approaches to understand the role of the root microbiome in plants. Schematic representation of information processing strategies (top-down and bottom-up) and the approaches that stand on the edges of them (reductionist and holistic). Experimentation complexity increases when more microorganisms are added to the investigation, whereas traits and member certainty decrease. Reductionist approaches tend to rely on causality analyzes, whereas holistic approaches most likely rely on correlational analyzes. SynCom experimentation lies in the middle ground, allowing a better understanding of microbe–microbe interactions.

As mentioned previously, research and scientific foundations to date have been developed using reductionist or holistic strategies, and as the more information emerges, the more interesting it is to fill the gaps between these two approaches. The use of SynComs arises as a promising “middle-out” point of view that can narrow the gap between the knowledge obtained with single strains and whole microbiomes (Figure 4). In general, experimentation complexity and certainty of the interacting factors are inversely proportional; whereas the first tend to increase as more strains are added to the research, the second one is bigger when fewer strains are studied. Moreover, correlational and causality analyses also increase and decrease inversely (Figure 4). While some researches focus on attaining specific traits within the community (Moronta-Barrios et al., 2018) or look for phylogenetic proximity (Burghardt et al., 2018; De Vrieze et al., 2018; Gadhave et al., 2018), others try to design SynComs by mixing different strains that differ either in their phylogenetic identity (Niu et al., 2017; Voges et al., 2019), origin of isolation (de Souza et al., 2019; Zhang et al., 2019), synthesis, emission, and/or response to volatile (Schulz-Bohm et al., 2015) and nonvolatile metabolites (Lebeis et al., 2015), or response patterns to root exudates (Herrera Paredes et al., 2018).

### The Whole of a Small SynCom Is Usually More Than the Sum of Its Parts

When the research aimed to evaluate PGPR traits, SynComs tended to be smaller, prioritizing a functional examination rather than an ecological analysis of the communities, looking for a simplified way to add the “community factor” or the interactions that can cause different outcomes. Employing a top-down approach, Zhuang et al. (2020) explored the wild bacterial community of the garlic rhizosphere to screen PGPR strains that also had effects on radish seedlings (*Raphanus sativus*). They found that increasing *Pseudomonas* community richness is beneficial to the plant biomass and nutrient content and, compared with single-strain inoculants, multistrain microbial inoculants can promote plant growth more reliably and effectively.

Small SynComs have also been used to assess the community impact in other PGPR traits such as biocontrol and host–immune response modulation. Lee et al. (2020) analyzed the microbial community structure of diseased and healthy tomato plants, detecting that the abundance of Gram-positive Actinobacteria and Firmicutes phyla was different, depending on the infection. They designed a SynCom with four Gram-positive species, which did not directly antagonize the pathogen, but displayed greater immune activation against it. Interestingly, plant protection was longer with the SynCom compared to each of the individual strains (Lee et al., 2020).

Based on the native community of *N. attenuata*, Santhanam et al. (2019) designed a five-strain SynCom with known biocontrol mechanisms. They concluded that the complementary abilities of the SynCom members account for the desired protective effect as only the consortium and not the single isolates or smaller combinations of them were able to significantly reduce the disease incidence caused by *Fusarium–Alternaria* phytopathogens in field-grown plants (Santhanam et al., 2015, 2019). Moreover, Niu et al. (2017) assembled a greatly simplified but representative seven-member SynCom that exerted a biocontrol effect over a pathogenic fungus that causes maize seedling blight, where the plants treated with the SynCom displayed the lowest disease severity index. Although each one of the seven community members showed some biocontrol effects against *Fusarium verticillioides*, the effects exhibited by the entire community were more substantial (Niu et al., 2017).

Small SynComs have also been used to study communication along the rhizosphere through the release of different chemicals. Schulz-Bohm et al. (2015) developed a soil model system mimicking more closely the natural context around the rhizosphere to understand the ecological role of volatiles in soil microbial interactions. They detected that a different blend of volatile organic compounds (VOCs; alcohols, ketones, esters, aromatic and organosulfur compounds, among others) was produced by a bacterial mixture of five strains, after comparison with the VOC profile of monocultures *in vitro*, and that some volatiles were only emitted by the bacterial mixture and not by the monocultures (Schulz-Bohm et al., 2015). Likewise, some antifungal compounds are only produced in diverse bacterial communities, suggesting that less abundant taxa play an important role in antifungal volatile production (Hol et al., 2015). However, pathogen-suppressive characteristics can be lost if stress exposure leads to an alteration of the community structure (van Agtmaal et al., 2015), effects that can be observed through the analysis of soil VOCs as a function of varying environmental factors over large spatiotemporal scales (McNeal and Herbert, 2009; van Agtmaal et al., 2015).

In this context, but not in the case of VOCs, Sánchez-Gorostiaga et al. (2019) observed that the contribution of a given species or pair of species to a community function may depend on the presence or absence of other taxa and that the effects are not always additive. Specifically, using mathematical modeling and *in vitro* experiments, they examined how the amylolytic rate of combinatorial assemblages of six starch-degrading soil bacteria depended on the separate functional contributions from each species and their interactions. They found that the ability of the model to predict community function declined as more species were added to the consortia (Sánchez-Gorostiaga et al., 2019), highlighting how complex and challenging it is to predict the functional interactions in microbial communities in bottom-up approaches.

### Microbe–Microbe Interactions as a Driver of a SynCom Outcome

Durán et al. (2018), using large SynComs (190 members) and a network inference tool to analyze the relationships of operational taxonomic units, revealed that negative interactions dominated between kingdoms (fungi, oomycetes, and bacteria), whereas within kingdoms, positive interactions were more frequent. Moreover, the absence or presence of certain groups (i.e., the bacterial community) had a critical impact on plant growth and health (Durán et al., 2018).

Selection and competition in host-associated bacterial populations influence rhizobial fitness, which can be explained in part by the host genotype. The symbiotic mutualistic relationship between nitrogen-fixing bacteria, such as *Ensifer meliloti*, and its legume plant hosts has been well-studied in single-strain experiments (Kraiser et al., 2011). However, little is known about the consequences of competition among strains over fitness in the rhizosphere. Burghardt et al. (2018) set out mixed-strain experiments to evaluate the strength of selection in multistrain competitive environments and estimate the resulting relative strain fitness. Host selection was dependent on the competition among strains, and the more efficient rhizobacteria (i.e., fixing nitrogen and/or contributing to host biomass) were more competitive at forming nodules or being rewarded by hosts (Burghardt et al., 2018).

### SynComs Value to Get Insights on Plant–Microbe Interactions

SynCom applications to directly study plant–microbe interactions have proved fruitful. Moccia et al. (2020) reported not only the existence of plant-driven influence over colonization of some bacterial species, but also several colonization patterns depending on the plant nutrient conditions, or the presence of some microorganisms, discovered through “drop-out communities” assays. The latter is based on the use of different SynComs that comprised the same members, with the difference that in each of the SynCom tested, one member or taxa has been excluded or “dropped out” (Moccia et al., 2020). Comparing drop-out communities with the corresponding full SynCom provides an opportunity to determine the individual effects each taxon may have on either the community or the plant host function (Niu et al., 2017; Finkel et al., 2019; Moccia et al., 2020).

Plant hormones, such as salicylic acid, are relevant agents in microbial colonization of different plant tissues, as shown by Lebeis et al. (2015). Plant immune system participation is vital to selectively allow colonization of beneficial nonpathogenic microbes while excluding potential pathogens. Their results showed that this phytohormone is required to assemble a normal root microbiome. The application of SynComs revealed that plant immune signaling drives the selection from available microbial communities to shape the root microbiome (Lebeis et al., 2015).

Microbiota recruitment can also differ among plant varieties. Zhang et al. (2019) observed a difference in nitrogen-use efficiency between two rice varieties (*indica* and *japonica*). After profiling their microbiomes and using genetic approaches, researchers found a nitrogen transporter and sensor (*NRT1.1B*) associated with the recruitment of a large proportion of *indica*-enriched bacteria (variety with the highest N-use efficiency) and designed bacterial SynComs based on *indica*- or *japonica*-enriched operational taxonomic units that were associated with *NRT1.1B*. Finally, they found that an *indica*-enriched SynCom had a larger effect on rice growth (Zhang et al., 2019).

Concerning plant–microbe interactions, several molecules, mainly plant secondary metabolites, are involved in shaping the microbiome structure. Voges et al. (2019) looked for community shifts that could occur in the absence of plant-secreted specialized small molecules, such as phytoalexins, flavonoids, and coumarins, employing an *A. thaliana* root-derived bacterial SynCom and plant mutant lines. They observed that a lack of coumarin caused a shift in the root microbial community, specifically under iron deficiency (Voges et al., 2019). It has been shown that these compounds alter the root microbiota composition and are required for microbiota-mediated plant iron uptake and immune regulation, contributing to a beneficial plant–microbiota interaction (Harbort et al., 2020). Regarding the effects of nutrient conditions on plant–microbe interactions, such as phosphate stress or starvation, Finkel et al. (2019) used large SynComs (185 strain members) to investigate how inorganic orthophosphate deficiency influences microbiome structure and, in turn, how this can affect plant health. They found that phosphate-stressed plants are susceptible to colonization by latent opportunistic competitors, thus exacerbating the plant's inorganic phosphate starvation condition (Finkel et al., 2019).

The same SynCom was used in Finkel et al. (2020), where the authors worked with four modules of coexisting microbes, to assess how the interaction between microorganisms influences the *Arabidopsis* root growth. Interestingly, they found that a single genus, *Variovorax*, was responsible for maintaining the plant root growth program, overriding the drastic inhibition of root growth that was induced by a wide diversity of bacterial strains and by the entire community. Furthermore, they found that a single operon for auxin degradation was necessary and sufficient to observe the effect of these *Variovorax* strains in roots and that this genus is among a limited group of core bacterial genera found in 30 different plant species, suggesting its vital role in the bacteria–bacteria–plant communication networks (Finkel et al., 2020).

### SynCom Design and Assessment: Context and Guidelines for Comparable Experiments

Not much vocabulary standardization was observed in the revised literature; terms like consortia, inocula, enrichment, community, and others were frequently used rather vaguely (definition of these and other terms is provided or suggested in Supplementary Table 3). We propose using the term “SynCom” when three or more isolates are used, each member of the consortium is well-known, each member is traceable (by any means), and the community is designed to address a specific research question. Additionally, we suggest choosing the term *in vivo* or *in planta* when SynComs are inoculated in plants growing in any substrate or cultivation system.

SynCom sizes were quite variable: a small consortium (up to 10 strains) may not be as taxonomically representative as a large one (>100 strains). However, it could be as functionally diverse to allow these communities' normal ecological development (Toju et al., 2018). Species-rich communities are often more efficient and more productive than species-poor communities as they use limited resources more efficiently (Menéndez and Paço, 2020).

For some studies, SynComs are designed to represent the core microbiome (Bulgarelli et al., 2012, 2013; Lundberg et al., 2012) of a given plant (Kong et al., 2018), but it is important to note that satellite microbes, that is, changing microorganisms not generally found in the core microbiome, possibly play critical modulatory roles under particular environmental conditions (Shi et al., 2016; Compant et al., 2019; O'Banion et al., 2020). Therefore, as low relative abundance microbes may play significant roles in plant ecosystem functioning (Schulz-Bohm et al., 2015), less represented species should not go unnoticed when designing a SynCom.

Several aspects concerning SynCom structure and dynamics should deserve some attention. Compatibility and antagonistic interactions between species were not tested in all analyzed SynCom reports (Moccia et al., 2020; Toju et al., 2020). Additionally, the starting inoculum concentration may influence the competitivity of the strains. Consequently, it would be more appropriate to test *in vitro* compatibility between strains (using different culture media), assessing the proper cell dosage before performing subsequent assays. Moreover, existing commercial isolates, which many laboratories use to mimic the WildCom of a given plant, might be genotypically or functionally distinct from microorganisms that researchers find in their new soil samples, thus not genuinely reflecting interactions between plants and the root microbiome in a given natural soil (Liu et al., 2019). Only some reports describe the assessment of antagonism among strains in compatibility tests consisting of small experiments such as the cross-streak method, spot inoculation in the vicinity, and evaluation of culture supernatants, among others (Vijayakumar and Muriana, 2015; Moran et al., 2016; Ijaz et al., 2019; Ishizawa et al., 2019; Olanrewaju and Babalola, 2019). Some authors claim they do not perform compatibility tests when members come from the same plant's natural environment. Nevertheless, having the same origin does not mean that the community members coexist without competition or antagonism.

The metabolomic profile of a given community depends on the interactions among its members, for example, a different VOC profile is found for single bacterial strains (Ledger et al., 2016) compared to bacterial mixtures, a profile that may be susceptible to the presence or absence of some nonabundant strains (Schulz-Bohm et al., 2015). In this context, auxotrophic interactions may allow auxotrophs—unable to synthesize particular compounds required for their growth—to exist and colonize a niche, thanks to the coexistence with other species (Johnson et al., 2020). On the same line, genes related to biosynthesis can be absent in auxotrophic microbes, but genes encoding for proteins involved in the efficient transport of specific metabolites may be present instead (Jiang et al., 2018). Likewise, specific plant growth–promoting traits present in some species may be an indirect consequence of functions in other microbes that allow these species to develop beneficial metabolites for the plant. In other words, the contribution of a given species or pair of species to a community function may also depend on the presence or absence of other taxa (Jiang et al., 2018; Sánchez-Gorostiaga et al., 2019).

A pivotal issue to assess the effects or performance of SynCom is to track the presence of these members in the plant study system. Unfortunately, not all applications of SynComs employed some method to follow up on the presence and persistence of the strains until the end of the experiment (Sánchez-Gorostiaga et al., 2019). Therefore, it is not always correct to link the observed effects with SynCom member functions. Several techniques improve the chances to perform a specific follow-up of different strains in an experiment, such as biomarkers, labels (i.e., tagged genes for further detection through spectrophotometry or microscopy), and advances in three-dimensional images with confocal microscopy (O'Banion et al., 2020). On the other hand, comparative studies using plant or microorganism mutants, or transgenic lines where gain and loss of function are tested, have also been especially useful to characterize different traits present in distinct species, giving a better understanding of microbial functioning or evolution (Zúñiga et al., 2013; Allard-Massicotte et al., 2016; Poupin et al., 2016).

Another factor to have in consideration is both the short- and long-term scales of the experiments. This is because proliferation, abundance, and population densities within the microbiome are in constant control and modulation across the plant life cycle (Chaparro et al., 2014), even in small time scales (Staley et al., 2017; Hubbard et al., 2018). Besides, time scales should be considered, as the order of species' arrival to the niche can result in significant differences in the mature community structure. Early colonizers often have an advantage because they can use resources before other microorganisms' appearance or because they can produce physical barriers or antibiotics that slow the colonization of subsequent microorganisms (Hu et al., 2020). Thus, in general, microbial species introduced early into communities are more likely to persist, controlling the assembly of latecomer species (Werner and Kiers, 2015; Sprockett et al., 2018; Toju et al., 2020).

Finally, a proper plant model should be selected, depending on the purpose of the study. If the aim is to explore the relationship of the plant ontogeny with the microbiota, an already well-described plant should be appropriate (Chaparro et al., 2013, 2014). For agronomical purposes, the yield of the crop might be considered. If the environmental effects are being evaluated, such as abiotic stress caused by saline, poor, acidic, or alkaline soils, one might want to explore the holobiont of native plants from these environments. When the aim is to study the underlying molecular mechanisms, it is better to connect this knowledge with plant genetics, biochemistry, metabolism, and molecular physiology (Poupin et al., 2013, 2016; Lebeis et al., 2015; Ledger et al., 2016; Finkel et al., 2019). Finally, if the aim is to study the microbiota solely as a biological community, comparative studies may be suitable (Zhang et al., 2019), and sometimes employing a plant model may not be essential. Here, high-throughput technologies, bioinformatics, and predictive models of microbial samples associated with plants can replace or complement plant-growth systems.

## Conclusion and Future Prospects

Not enough is known about plant–microbe interactions and even less about microbe–microbe interactions in the plant rhizosphere. The development and employment of SynComs are giving more insights into the rhizosphere dynamics and structure, and how these dynamics influence plant fitness and behavior. Nevertheless, more systematic and standardized investigations are needed to harness the full potential of this approach or to perform statistical meta-analyses. Over time, experimental approaches may incorporate even more factors and complexity into the system, such as spatiotemporal analyses and ecological dynamics, and may include more taxonomic levels to the interactions. Current research on Archea and Eukarya kingdoms is emerging; and as more individuals are being sequenced, more protists—key microbiome predators—are also being linked to the plant holobiont. Finally, better tracking systems to consider, for instance, the auxotrophy phenomena or the rapid community fluctuations, and the use of computational and mathematical modeling will also bring additional insights and tools to uncover the still-hidden mysteries of the rhizosphere.

## Data Availability Statement

The original contributions presented in the study are included in the article/Supplementary Material, further inquiries can be directed to the corresponding author/s.

## Author Contributions

MP and OM conceived the idea and performed the analyses. All authors drafted and commented on the manuscript.

## Conflict of Interest

The authors declare that the research was conducted in the absence of any commercial or financial relationships that could be construed as a potential conflict of interest.

## Supplementary Material

Supplementary Figure 1 PRISMA diagram of the literature filtering process.

Supplementary Table 1 Overview of the published articles analyzed in this systematic review, organized in three SynCom size categories (small, medium, and large). For each article, the title, keywords (if any), main highlights, plant model, SynCom size, and the respective references are provided.

Supplementary Table 2 Compilation of the bacterial isolates and their taxonomic classifications.

Supplementary Table 3 Guideline for term usage related to this topic.
