# Supplementary material for: From Microbial Dynamics to Functionality in the Rhizosphere: A Systematic Review of the Opportunities With Synthetic Microbial Communities
Source: Front Plant Sci. 2021 Jun 3;12:650609. doi: 10.3389/fpls.2021.650609 (PMC8210828; doi:10.3389/fpls.2021.650609)
Supplement: Supplementary Figure 1 — PRISMA diagram of the literature filtering process. [file Data_Sheet_1.zip › Supplementary Table 1.docx]

**Supplementary Table 1**. Overview of published articles analyzed in this systematic review, organized in three SynCom size categories (small, medium and large). For each published article, the title, keywords (if any), main highlights, plant model, SynCom size and reference is shown.

|  | **Title** | **Keywords** | **Highlights** | **Plant model** | **SynCom size** | **Reference** |
| --- | --- | --- | --- | --- | --- | --- |
| **SMALL SynComs (up to ten members)** | Combining Different Potato-Associated *Pseudomonas* Strains for Improved Biocontrol of *Phytophthora infestans* | Solanum tuberosum; biocontrol; consortium; late blight; phyllosphere; pseudomonads; rhizosphere. | Two strains whose dual combination was efficient were weakly interfering with each other’s growth and had complementary modes of action.  Small communities can provide more protection than the application of single strains. | *Solanum tuberosum* | 3 | (De Vrieze et al., 2018) |
|  | Combined application of biochar and PGPR consortia for sustainable production of wheat under semiarid conditions with a reduced dose of synthetic fertilizer | biochar; economic returns; grain yield; Nitrogen fixation; PGPR. | The use of the PGPR consortia and biochar can improve the yield and profit of wheat crop with reduced synthetic fertilization. | *Triticum aestivum* | 3 | (Ijaz et al., 2019) |
|  | Soil Inoculation with *Bacillus* spp. Modifies Root Endophytic Bacterial Diversity, Evenness, and Community Composition in a Context-Specific Manner | 454 pyrosequencing; Bacillus; endophytes; microbial inoculants; plant growth-promoting rhizobacteria. | The Bacillus inocula themselves failed to successfully colonize the endosphere; however, the effects they extended on the endophytic bacterial community were both generic as well as species specific. | *Brassica oleracea* | 3 | (Gadhave et al., 2018) |
|  | Deciphering composition and function of the root microbiome of a legume plant | clover, root, microbiome, 16S rRNA sequencing, microcosm | The negative effect of an abundant root microbiome member when inoculated alone (reduced plant growth) was alleviated if it was co-inoculated with other root microbiome members. | *Trifolium pratense* | 4 | (Hartman et al., 2017) |
|  | **Title** | **Keywords** | **Highlights** | **Plant model** | **SynCom size** | **Reference** |
| **SMALL SynComs (up to ten members)** | Disruption of Firmicutes and Actinobacteria abundance in tomato rhizosphere causes the incidence of bacterial wilt disease | - | Dysbiosis of a protective bacterial community in diseased rhizosphere soil (DRS) promotes the incidence of disease.  Four strains did not directly antagonize R. solanacearum but activated plant immunity. | *Solanum lycopersicum* | 4 | (Lee et al., 2020) |
|  | A suite of complementary biocontrol traits allows a native consortium of root-associated bacteria to protect their host plant from a fungal sudden-wilt disease | *Nicotiana attenuata*; antibiosis; bacterial consortium; biocontrol; biofilm formation; niche colonization; nutrient competition; sudden wilt disease. | Five-member bacterial consortium protects  its host against fungal phytopathogens via complementary traits. | *Nicotiana attenuata* | 5 | (Santhanam et al., 2019) |
|  | Synthetic community with six *Pseudomonas* strains screened from garlic rhizosphere microbiome promotes plant growth | - | Pseudomonas is a key PGPR genus  in the rhizosphere of garlic. | *Allium sativum* | 6 | (Zhuang et al., 2020) |
|  | High-order interactions distort the functional landscape of microbial consortia | - | A quantitative framework that allows to separate the effect of behavioral and population dynamics interactions is proposed. | *No plant* | 6 | (Sánchez-Gorostiaga et al., 2019) |
|  | **Title** | **Keywords** | **Highlights** | **Plant model** | **SynCom size** | **Reference** |
| **SMALL SynComs (up to ten members)** | Pathogen-induced activation of disease-suppressive functions in the endophytic root microbiome | - | Consortium of Chitinophaga and Flavobacterium consistently suppressed fungal root disease. A previously unidentified NRPS-PKS gene cluster from Flavobacterium was essential for disease suppression by the endophytic consortium. | *Beta vulgaris* | 7 | (Carrión et al., 2019) |
|  | Quantification of the Composition Dynamics of a Maize Root-associated Simplified Bacterial Community and Evaluation of Its Biological Control Effect | community assembly; dynamics; maize; selective medium; synthetic community; biological control. | A seven-species synthetic community representing the root microbiome has the potential to serve as a useful system to explore how bacterial interspecies interactions affect root microbiome assembly and to dissect the beneficial effects of the root microbiota on hosts.  The removal of E. cloacae led to the complete loss of the community; suggesting that E. cloacae plays the role of keystone species in this model ecosystem. | *Zea mays* | 7 | (Niu et al., 2017) |
|  | Straw biochar increases the abundance of inorganic phosphate solubilizing bacterial community for better rape (*Brassica napus*) growth and phosphate uptake | Brassica napus; inorganic phosphate solubilizing bacteria; phosphate uptake; Phosphorus; straw biochar. | Synthetic inorganic-phosphate-solubilizing bacteria (iPSB) community had a larger capacity to solubilize inorganic P and exude organic anions than any of the individual strains, and its abundance was significantly correlated with plant biomass, P content and P uptake. | *Brassica napus* | 7 | (Zheng et al., 2019) |
| **SMALL SynComs (up to ten members)** | Probiotic Diversity Enhances Rhizosphere Microbiome Function and Plant Disease Suppression | - | The survival of introduced Pseudomonas consortia increased with increasing diversity, and the SynCom reduced pathogen density in the rhizosphere and decreased the disease incidence due to both intensified resource competition and interference with the pathogen. | *Solanum lycopersicum* | 8 | (Hu et al., 2016) |
|  | Distinguishing nutrient-dependent plant driven bacterial colonization patterns in alfalfa | - | Unique, repeatable colonization phenotypes for individual microbes during plant microbiome assembly, whose alterations are caused by the host plant, microbes, and available nutrients. | *Medicago sativa* | 8 | (Moccia et al., 2020) |
|  | Bacterial Microbiota of Rice Roots: 16S-Based Taxonomic Profiling of Endophytic and Rhizospheric Diversity, Endophytes Isolation and Simplified Endophytic Community | core plant microbiome; endophyte; plant microbiome; | There is a possible colonization cooperation between species of the simplified community that enabled to form a stable multispecies community. | *Oryza sativa* | 10 | (Moronta-Barrios et al., 2018) |
|  | A fragrant neighborhood: volatile mediated bacterial interactions in soil | inter-specific interactions; low-abundant bacteria; rhizosphere; soil microcosms; synthetic microbial communities; volatiles. | Microbial interactions and shifts in microbial community composition had a strong effect on the volatile emission.  Volatiles produced during microbial interactions in the rhizosphere have a significant long-distance effect on microorganisms in the surrounding, nutrient-depleted soil. | *No plant* | 10 | (Schulz-Bohm et al., 2015) |
|  | **Title** | **Keywords** | **Highlights** | **Plant model** | **SynCom size** | **Reference** |
| **MEDIUM SynComs (between 11 and 100 members)** | Genome Sequences of a Plant Beneficial Synthetic Bacterial Community Reveal Genetic Features for Successful Plant Colonization | bacterial genome; plant microbiome; plant-beneficial bacteria; plant–microbe association; synthetic microbial community (SynCom) | Diversification of the nutrient uptake phenotypes in bacteria could be a determinant for successful bacterial colonization of plants. | *Zea mays* | 17 | (de Souza et al., 2019) |
|  | A Community-Based Culture Collection for Targeting Novel Plant Growth-Promoting Bacteria from the Sugarcane Microbiome | community-based culture collection (CBC); maize; microbiome; plant growth-promoting (PGP); sugarcane; synthetic community. | Abundance-based synthetic inoculants can be successfully applied to recover beneficial plant microbes from plant microbiota. | *Zea mays* | 20 | (Armanhi et al., 2018) |
|  | NRT1.1B is associated with root microbiota composition and nitrogen use in field-grown rice | - | Rice nitrate transporter and sensor, NRT1.1B, is associated with the recruitment of a large proportion of root-associated bacteria. | *Oryza sativa* | 20 | (Zhang et al., 2019) |
|  | Plant-derived coumarins shape the composition of an *Arabidopsis* synthetic root microbiome | coumarins; plant microbiome; plant specialized metabolism; synthetic communities | Elucidation of specific mechanisms by which plant-derived molecules mediate microbial community composition through a systematic approach. | *Arabidopsis thaliana* | 22 | (Voges et al., 2019) |
|  | Rhizosphere-enriched microbes as a pool to design synthetic communities for reproducible beneficial outputs | fusarium wilt; growth promotion; rhizosphere competitive traits; suppressiveness; synthetic communities; tomato | Application of SynComs on poor substrates can yield reproducible plant phenotypes. | *Arabidopsis thaliana* | 25 | (Tsolakidou et al., 2019) |
|  | **Title** | **Keywords** | **Highlights** | **Plant model** | **SynCom size** | **Reference** |
| **MEDIUM SynComs (between 11 and 100)** | Rhizosphere microbiome functional diversity and pathogen invasion resistance build up during plant development | - | High functional diversity of ‘climax’ microbiomes leads to a better resistance to bacterial pathogen invasion.  Community-level processes may supersede the importance  of single species. | *Solanum lycopersicum* | 32 | (Hu et al., 2020) |
|  | Root microbiota drive direct integration of phosphate stress and immunity | - | A genetic network controlling phosphate stress response influences root microbiome community structure.  Transcriptional regulators of phosphate stress response in Arabidopsis also directly repress defense. | *Arabidopsis thaliana* | 35 | (Castrillo et al., 2017) |
|  | Salicylic acid modulates colonization of the root microbiome by specific bacterial taxa | - | Biosynthesis and signaling dependent on salicylic acid is required to assemble a normal root microbiome, whose colonization is modulated by specific bacterial families. | *Arabidopsis thaliana* | 38 | (Lebeis et al., 2015) |
|  | Design of synthetic bacterial communities for predictable plant phenotypes | - | It is possible to infer causal relationships between microbiota membership and host phenotypes and to use these inferences to rationally design novel communities. | *Arabidopsis thaliana* | 84 | (Herrera Paredes et al., 2018) |
|  | **Title** | **Keywords** | **Highlights** | **Plant model** | **SynCom size** | **Reference** |
| **LARGE SynComs (more than 100 members)** | Select and resequence reveals relative fitness of bacteria in symbiotic and free-living environments | *Ensifer meliloti*; Medicago; evolve and resequence; facultative mutualism; synthetic community. | Selection that hosts exert on rhizobial communities depends on competition among strains.  The greatest changes in allele frequencies in response to plant hosts are in genes associated with motility, regulation of nitrogen fixation, and host/rhizobia signaling. | *No plant* | 101 | (Burghardt et al., 2018) |
|  | The effects of soil phosphorus content on plant microbiota are driven by the plant phosphate starvation response | - | Pi-stressed plants are susceptible to colonization by latent opportunistic competitors found within their microbiome, thus exacerbating the plant’s Pi starvation. | *No plant* | 185 | (Finkel et al., 2019) |
|  | A single bacterial genus maintains root growth in a complex microbiome | - | Genus Variovorax reverses inhibition of root growth induced by bacterial strains.  Metabolic signal interference shapes bacteria–plant communication networks, essential for maintaining normal growth. | *Arabidopsis thaliana* | 185 | (Finkel et al., 2020) |
|  | Root Microbiome Modulates Plant Growth Promotion Induced by Low Doses of Glyphosate | glyphosate; hormesis; microbiome | Glyphosate hormesis was completely dependent on the root microbiome composition, specifically on the presence of root growth inhibitor strains. | *Arabidopsis thaliana* | 185 | (Ramirez-Villacis et al., 2020) |
|  | **Title** | **Keywords** | **Highlights** | **Plant model** | **SynCom size** | **Reference** |
| **LARGE SynComs (more than 100 members)** | Functional overlap of the *Arabidopsis* leaf and root microbiota | - | An extensive taxonomic overlap between the leaf and root microbiota was found. Through defined bacterial communities, it was shown that the isolates form assemblies resembling natural microbiota and are also capable of ectopic leaf or root colonization. | *Arabidopsis thaliana* | 188 | (Bai et al., 2015) |
|  | Microbial Interkingdom Interactions in Roots Promote *Arabidopsis* Survival | bacteria; fungi; microbe-microbe interactions; oomycetes; plant microbiota; synthetic microbial communities. | The bacterial microbiota is essential for plant survival and protection against root-derived filamentous eukaryotes.  Biocontrol activity of bacterial root commensals is a redundant trait that maintains microbial interkingdom balance for plant health. | *Arabidopsis thaliana* | 190 | (Durán et al., 2018) |

**References**

Armanhi, J. S. L., de Souza, R. S. C., Damasceno, N. de B., de Araújo, L. M., Imperial, J., & Arruda, P. (2018). A community-based culture collection for targeting novel plant growth-promoting bacteria from the sugarcane microbiome. *Frontiers in Plant Science*, *8*(January), 1–17. https://doi.org/10.3389/fpls.2017.02191

Bai, Y., Müller, D. B., Srinivas, G., Garrido-Oter, R., Potthoff, E., Rott, M., Dombrowski, N., Münch, P. C., Spaepen, S., Remus-Emsermann, M., Hüttel, B., McHardy, A. C., Vorholt, J. A., & Schulze-Lefert, P. (2015). Functional overlap of the Arabidopsis leaf and root microbiota. *Nature*, *528*(7582), 364–369. https://doi.org/10.1038/nature16192

Burghardt, L. T., Epstein, B., Guhlin, J., Nelson, M. S., Taylor, M. R., Young, N. D., Sadowsky, M. J., & Tiffin, P. (2018). Select and resequence reveals relative fitness of bacteria in symbiotic and free-living environments. *Proceedings of the National Academy of Sciences of the United States of America*, *115*(10), 2425–2430. https://doi.org/10.1073/pnas.1714246115

Carrión, V. J., Perez-Jaramillo, J., Cordovez, V., Tracanna, V., De Hollander, M., Ruiz-Buck, D., Mendes, L. W., van Ijcken, W. F. J., Gomez-Exposito, R., Elsayed, S. S., Mohanraju, P., Arifah, A., van der Oost, J., Paulson, J. N., Mendes, R., van Wezel, G. P., Medema, M. H., & Raaijmakers, J. M. (2019). Pathogen-induced activation of disease-suppressive functions in the endophytic root microbiome. *Science*, *366*(6465), 606–612. https://doi.org/10.1126/science.aaw9285

Castrillo, G., Teixeira, P. J. P. L., Paredes, S. H., Law, T. F., De Lorenzo, L., Feltcher, M. E., Finkel, O. M., Breakfield, N. W., Mieczkowski, P., Jones, C. D., Paz-Ares, J., & Dangl, J. L. (2017). Root microbiota drive direct integration of phosphate stress and immunity. *Nature*, *543*(7646), 513–518. https://doi.org/10.1038/nature21417

de Souza, R. S. C., Armanhi, J. S. L., Damasceno, N. de B., Imperial, J., & Arruda, P. (2019). Genome Sequences of a Plant Beneficial Synthetic Bacterial Community Reveal Genetic Features for Successful Plant Colonization. *Frontiers in Microbiology*, *10*(August). https://doi.org/10.3389/fmicb.2019.01779

De Vrieze, M., Germanier, F., Vuille, N., & Weisskopf, L. (2018). Combining Different Potato-Associated Pseudomonas Strains for Improved Biocontrol of Phytophthora infestans. *Frontiers in Microbiology*, *9*(October), 1–13. https://doi.org/10.3389/fmicb.2018.02573

Durán, P., Thiergart, T., Garrido-Oter, R., Agler, M., Kemen, E., Schulze-Lefert, P., & Hacquard, S. (2018). Microbial Interkingdom Interactions in Roots Promote Arabidopsis Survival. *Cell*, *175*(4), 973-983.e14. https://doi.org/10.1016/j.cell.2018.10.020

Finkel, O. M., Salas-González, I., Castrillo, G., Conway, J. M., Law, T. F., Teixeira, P. J. P. L., Wilson, E. D., Fitzpatrick, C. R., Jones, C. D., & Dangl, J. L. (2020). A single bacterial genus maintains root growth in a complex microbiome. *Nature*. https://doi.org/10.1038/s41586-020-2778-7

Finkel, O. M., Salas-González, I., Castrillo, G., Spaepen, S., Law, T. F., Teixeira, P. J. P. L., Jones, C. D., & Dangl, J. L. (2019). The effects of soil phosphorus content on plant microbiota are driven by the plant phosphate starvation response. In *PLoS Biology* (Vol. 17, Issue 11). https://doi.org/10.1371/journal.pbio.3000534

Gadhave, K. R., Devlin, P. F., Ebertz, A., Ross, A., & Gange, A. C. (2018). Soil Inoculation with Bacillus spp. Modifies Root Endophytic Bacterial Diversity, Evenness, and Community Composition in a Context-Specific Manner. *Microbial Ecology*, *76*(3), 741–750. https://doi.org/10.1007/s00248-018-1160-x

Hartman, K., van der Heijden, M. G. A., Roussely-Provent, V., Walser, J. C., & Schlaeppi, K. (2017). Deciphering composition and function of the root microbiome of a legume plant. *Microbiome*, *5*(1), 1–13. https://doi.org/10.1186/s40168-016-0220-z

Herrera Paredes, S., Gao, T., Law, T. F., Finkel, O. M., Mucyn, T., Teixeira, P. J. P. L., Salas-González, I., Feltcher, M. E., Powers, M. J., Shank, E. A., Jones, C. D., Jojic, V., Dangl, J. L., & Castrillo, G. (2018). Design of synthetic bacterial communities for predictable plant phenotypes. In *PLoS Biology* (Vol. 16, Issue 2). https://doi.org/10.1371/journal.pbio.2003962

Hu, J., Wei, Z., Friman, V.-P. P., Gu, S. H., Wang, X. F., Eisenhauer, N., Yang, T. J., Ma, J., Shen, Q. R., Xu, Y. C., Jousseta, A., & Jousset, A. (2016). Probiotic Diversity Enhances Rhizosphere Microbiome Function and. *Mbio*, *7*(6), 1–8. https://doi.org/10.1128/mBio.01790-16.Editor

Hu, J., Wei, Z., Kowalchuk, G. A., Xu, Y., Shen, Q., & Jousset, A. (2020). Rhizosphere microbiome functional diversity and pathogen invasion resistance build up during plant development. *Environmental Microbiology*, *00*. https://doi.org/10.1111/1462-2920.15097

Ijaz, M., Tahir, M., Shahid, M., Ul-Allah, S., Sattar, A., Sher, A., Mahmood, K., & Hussain, M. (2019). Combined application of biochar and PGPR consortia for sustainable production of wheat under semiarid conditions with a reduced dose of synthetic fertilizer. *Brazilian Journal of Microbiology*, *50*(2), 449–458. https://doi.org/10.1007/s42770-019-00043-z

Lebeis, S. L., Paredes, S. H., Lundberg, D. S., Breakfield, N., Gehring, J., McDonald, M., Malfatti, S., Del Rio, T. G., Jones, C. D., Tringe, S. G., & Dangl, J. L. (2015). Salicylic acid modulates colonization of the root microbiome by specific bacterial taxa. *Science*, *349*(6250), 860–864. https://doi.org/10.1126/science.aaa8764

Lee, S. M., Kong, H. G., Song, G. C., & Ryu, C. M. (2020). Disruption of Firmicutes and Actinobacteria abundance in tomato rhizosphere causes the incidence of bacterial wilt disease. *ISME Journal*. https://doi.org/10.1038/s41396-020-00785-x

Moccia, K., Willems, A., Papoulis, S., Flores, A., Forister, M. L., Fordyce, J. A., & Lebeis, S. L. (2020). Distinguishing nutrient-dependent plant driven bacterial colonization patterns in alfalfa. *Environmental Microbiology Reports*, *12*(1), 70–77. https://doi.org/10.1111/1758-2229.12815

Moronta-Barrios, F., Gionechetti, F., Pallavicini, A., Marys, E., & Venturi, V. (2018). Bacterial Microbiota of Rice Roots: 16S-Based Taxonomic Profiling of Endophytic and Rhizospheric Diversity, Endophytes Isolation and Simplified Endophytic Community. *Microorganisms*, *6*(1), 14. https://doi.org/10.3390/microorganisms6010014

Niu, B., Paulson, J. N., Zheng, X., & Kolter, R. (2017). Simplified and representative bacterial community of maize roots. *Proceedings of the National Academy of Sciences of the United States of America*, *114*(12), E2450–E2459. https://doi.org/10.1073/pnas.1616148114

Ramirez-Villacis, D., Finkel, O. M., Salas-González, I., Fitzpatrick, C. R., Dangl, J. L., Jones, C. D., & Leon-Reyes, A. (2020). *Root Microbiome Modulates Plant Growth Promotion Induced by Low Doses of Glyphosate*. *July*, 1–11. https://doi.org/10.46678/pb.20.1383160

Sánchez-Gorostiaga, A., Bajić, D., Osborne, M. L., Poyatos, J. F., & Sanchez, A. (2019). High-order interactions distort the functional landscape of microbial consortia. In *PLoS Biology* (Vol. 17, Issue 12). https://doi.org/10.1371/journal.pbio.3000550

Santhanam, R., Menezes, R. C., Grabe, V., Li, D., Baldwin, I. T., & Groten, K. (2019). A suite of complementary biocontrol traits allows a native consortium of root-associated bacteria to protect their host plant from a fungal sudden-wilt disease. *Molecular Ecology*, *28*(5), 1154–1169. https://doi.org/10.1111/mec.15012

Schulz-Bohm, K., Zweers, H., de Boer, W., & Garbeva, P. (2015). A fragrant neighborhood: Volatile mediated bacterial interactions in soil. *Frontiers in Microbiology*, *6*(NOV), 1–11. https://doi.org/10.3389/fmicb.2015.01212

Tsolakidou, M. D., Stringlis, I. A., Fanega-Sleziak, N., Papageorgiou, S., Tsalakou, A., & Pantelides, I. S. (2019). Rhizosphere-enriched microbes as a pool to design synthetic communities for reproducible beneficial outputs. *FEMS Microbiology Ecology*, *95*(10), 1–14. https://doi.org/10.1093/femsec/fiz138

Voges, M. J. E. E. E., Bai, Y., Schulze-Lefert, P., & Sattely, E. S. (2019). Plant-derived coumarins shape the composition of an Arabidopsis synthetic root microbiome. *Proceedings of the National Academy of Sciences of the United States of America*, *116*(25), 12558–12565. https://doi.org/10.1073/pnas.1820691116

Zhang, J., Liu, Y. X., Zhang, N., Hu, B., Jin, T., Xu, H., Qin, Y., Yan, P., Zhang, X., Guo, X., Hui, J., Cao, S., Wang, X., Wang, C., Wang, H., Qu, B., Fan, G., Yuan, L., Garrido-Oter, R., … Bai, Y. (2019). NRT1.1B is associated with root microbiota composition and nitrogen use in field-grown rice. *Nature Biotechnology*, *37*(6), 676–684. https://doi.org/10.1038/s41587-019-0104-4

Zheng, B. X., Ding, K., Yang, X. R., Wadaan, M. A. M., Hozzein, W. N., Peñuelas, J., & Zhu, Y. G. (2019). Straw biochar increases the abundance of inorganic phosphate solubilizing bacterial community for better rape (Brassica napus) growth and phosphate uptake. *Science of the Total Environment*, *647*, 1113–1120. https://doi.org/10.1016/j.scitotenv.2018.07.454

Zhuang, L., Li, Y., Wang, Z., Yu, Y., Zhang, N., Yang, C., Zeng, Q., & Wang, Q. (2020). Synthetic community with six Pseudomonas strains screened from garlic rhizosphere microbiome promotes plant growth. *Microbial Biotechnology*, *202005210130046*. https://doi.org/10.1111/1751-7915.13640
