# Supplementary material for: From Microbial Dynamics to Functionality in the Rhizosphere: A Systematic Review of the Opportunities With Synthetic Microbial Communities
Source: Front Plant Sci. 2021 Jun 3;12:650609. doi: 10.3389/fpls.2021.650609 (PMC8210828; doi:10.3389/fpls.2021.650609)
Supplement: Supplementary Figure 1 — PRISMA diagram of the literature filtering process. [file Data_Sheet_1.zip › Supplementary Table 3.docx]

**Supplementary Table 3**. Guideline for term usage related to this topic.

| **Term** | **Definition** | **Reference**  **(if applicable)** |
| --- | --- | --- |
| **Inoculum**  ***plural: inocula*** | Any group of cells that are added to a sterile medium or to an already existing culture. |  |
| **Consortium**  ***plural: consortia*** | Any group of heterogeneous cells. |  |
| **Multi-strain bacterial consortium** | A group of different species of bacteria which act together as a community. |  |
| **Community** | Two or more populations that coexist in the same habitat and interact between each other. | (Audesirk et al., 2008) |
| **SynCom** | Group of 3 or more isolates that are consciously selected to assess a specific research objective. |  |
| **WildCom** | Microbial community of a given plant (or part of plant) in a given niche. |  |
| **Enriched community** | Community that is composed of populations already existing in the wild community in question. |  |
| ***In vivo* experiments** | When the effects are tested in the plant as a living organism. |  |
| **Core microbiome** | Sets of microorganisms that form cores of interactions that can be used to optimize microbial functions at the individual plant and ecosystem levels. They have high potential to organize microbiomes in ways that benefit host plants. | (Toju et al., 2018) |
| **Satellite microbiome** | Rare microbial taxa that occur in low abundance, not as representative as core microbes, defined on the basis of geographical range, local abundance, and habitat specificity. | (Compant et al., 2019) |
| **Hub microbes** | Species with key topological positions within the interaction network. | (Toju et al., 2018) |
| **Keystone species** | Species whose impacts on its community or ecosystem are large, and much larger than would be expected from its abundance. | (Cottee-Jones & Whittaker, 2012; Power & Mills, 1995) |

**References**

Audesirk, T., Audesirk, G., & Byers, B. E. (2008). Biología. La Vida en la Tierra. In *Biología. La vida en la tierra.*

Compant, S., Samad, A., Faist, H., & Sessitsch, A. (2019). A review on the plant microbiome: Ecology, functions, and emerging trends in microbial application. *Journal of Advanced Research*, *19*, 29–37. https://doi.org/10.1016/j.jare.2019.03.004

Cottee-Jones, H. E. W., & Whittaker, R. J. (2012). perspective: The keystone species concept: a critical appraisal. *Frontiers of Biogeography*, *4*(3). https://doi.org/10.21425/f5fbg12533

Power, M. E., & Scott Mills, L. (1995). The keystone cops meet in Hilo. *Trends in Ecology & Evolution*, *10*(5), 182–184. https://doi.org/10.1016/S0169-5347(00)89047-3

Toju, H., Peay, K. G., Yamamichi, M., Narisawa, K., Hiruma, K., Naito, K., Fukuda, S., Ushio, M., Nakaoka, S., Onoda, Y., Yoshida, K., Schlaeppi, K., Bai, Y., Sugiura, R., Ichihashi, Y., Minamisawa, K., & Kiers, E. T. (2018). Core microbiomes for sustainable agroecosystems. *Nature Plants*, *4*(5), 247–257. https://doi.org/10.1038/s41477-018-0139-4
