# Supplementary figures and images for: From Microbial Dynamics to Functionality in the Rhizosphere: A Systematic Review of the Opportunities With Synthetic Microbial Communities
Source: Front Plant Sci. 2021 Jun 3;12:650609. doi: 10.3389/fpls.2021.650609 (PMC8210828; doi:10.3389/fpls.2021.650609)

## PRISMA Flow Diagram of the literature filtering process

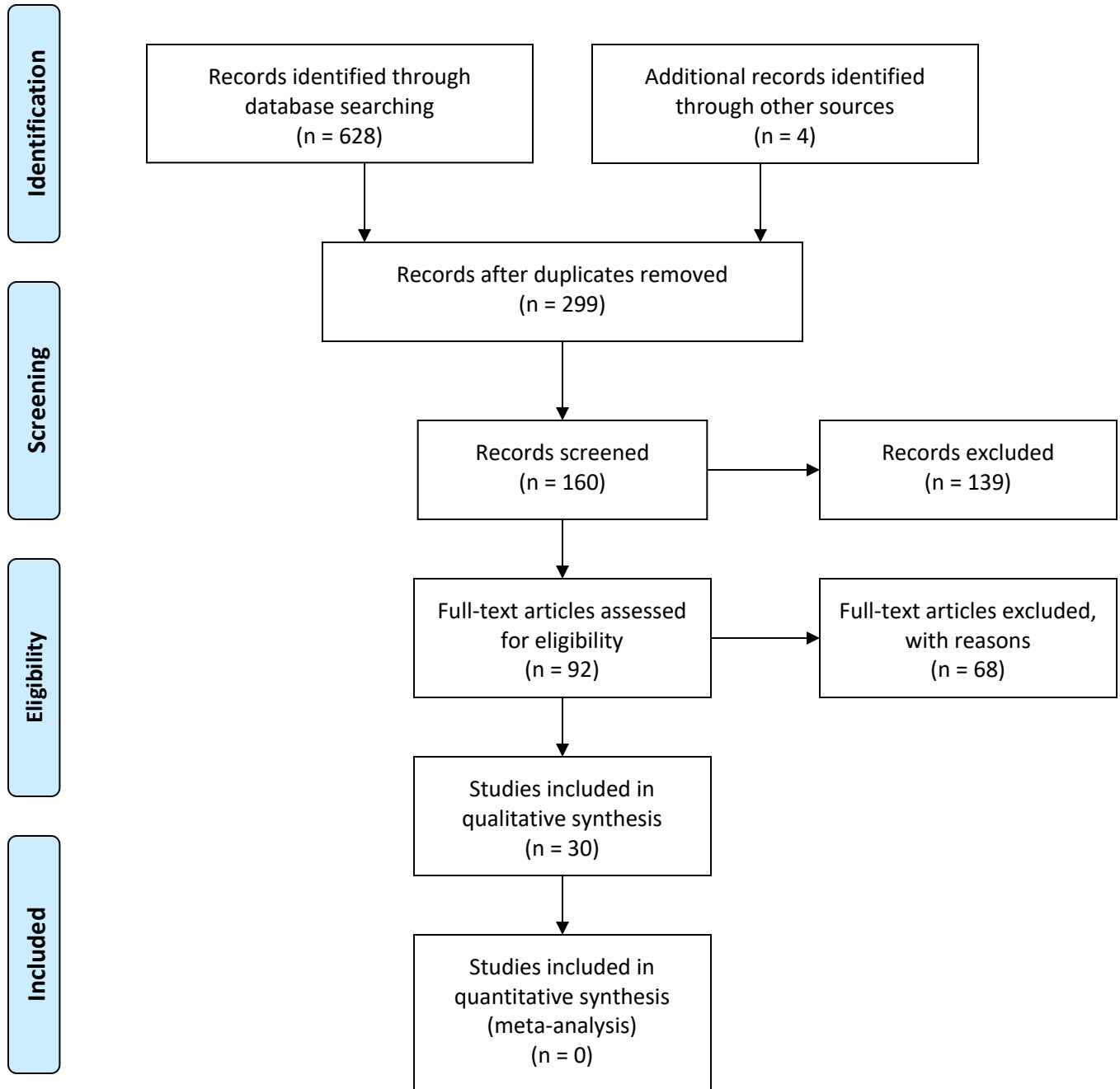

Supplement: Supplementary Figure 1 — PRISMA diagram of the literature filtering process. [file Data_Sheet_1.zip › Supplementary Figure 1.pdf]
